# A Snapshot of Microbial Succession and Volatile Compound Dynamics in Flat Peach Wine During Spontaneous Fermentation

**DOI:** 10.3389/fmicb.2022.919047

**Published:** 2022-06-29

**Authors:** Xiaoyu Xu, Yuanyuan Miao, Huan Wang, Piping Ye, Tian Li, Chunyan Li, Ruirui Zhao, Bin Wang, Xuewei Shi

**Affiliations:** Food College, Shihezi University, Shihezi, China

**Keywords:** flat peach, spontaneous fermentation, microbial communities, volatile composition, correlation analysis

## Abstract

Flat peaches possess characteristic flavors and are rich in nutrients. The fermentation of flat peaches to produce wine through complex biochemical reactions is an effective method to overcome their seasonal defects. Spontaneously fermented flat peach wine has plentiful and strong flavors, but the microbiota of fermentation are still unknown. In this study, the microbial succession and volatile compound dynamics of spontaneous fermentation in Xinjiang flat peach wine were investigated using high-throughput sequencing (HTS) and headspace solid phase microextraction (HS-SPME) coupled with gas chromatography-mass spectrometry (GC-MS) technology, respectively, to better understand the microbiota involved. Multivariate data analysis was used to predict the relationship between microorganisms and volatile chemicals. The results showed that *Kazachstania*, *Pichia*, *Aspergillus*, *Fructobacillus*, *Leuconostoc*, and *Lactobacillus* were the dominant genera during the spontaneous fermentation of flat peach wine. Furthermore, ethyl hexanoate, 3-hexen-1-yl acetate, ethyl caprate, ethyl caprylate, phenethyl acetate, ethanol, γ-decalactone, decanal, 1-hexanoic acid, and octanoic acid endued flat peach wine with a strong fruity and fatty aroma. The core functional microbiota (primarily consisting of 11 bacterial and 14 fungal taxa) was strongly associated with the production of 27 volatile compounds in the spontaneously fermented flat peach wine, according to multivariate data analysis. Some alcohols and esters were positively linked with the presence of *Kazachstania* and *Pichia*. Meanwhile, the presence of *Fructobacillus*, *Leuconostoc*, *Lactobacillus*, and *Weissella* was significantly correlated with 2-non-anol, ethanol, 3-methyl-1-butanol, octyl formate, isoamyl lactate, and ethyl lactate. This snapshot of microbial succession and volatile compound dynamics provides insights into the microorganisms involved in flat peach wine fermentation and could guide the production of flat peach wine with desirable characteristics.

## Introduction

Peaches [*Prunus persica* (L.) Batsch] originated in western China and have been widely cultivated in China for more than 3,000 years. Today, peaches are also cultivated in over 80 countries and regions worldwide (Li and Wang, 2020). Flat peaches [*Prunus persica* (L.) Batsch. *var. compressa* Bean], a special variety of peaches, are popular among customers due to their rich smell, delicious taste, and nutritious nature. Owing to its typical continental climate and ample light and heat availability, the large temperature difference between day and night, and long daylight hours, Xinjiang—located in the Eurasian continental bridge’s hinterland—offers considerable resource advantages for flat peach cultivation (Ma et al., 2002).

Flat peaches have become an important fruit from a consumer perspective. However, the flat peach is typical climacteric fruits, and its thin skin makes it extremely susceptible to mechanical damage and microbial infection during post-harvest transportation (Amoros et al., 1989). Therefore, it is necessary to develop different flat peach products to meet consumer and market needs. To this end, approaches such as physical preservation (e.g., irradiation treatment, pressure reduction treatment, air conditioning storage) (Fernández-Trujilio et al., 1998), chemical preservation (e.g., bioregulator treatment and calcium treatment) (Perkins-Veazie et al., 1999), and biological preservation (e.g., microbial preservation and preservation of natural extracted substances) (Fan et al., 2000) have been developed for peach preservation. However, these methods cannot fundamentally solve the problem of the post-harvest preservation of flat peaches. Therefore, other peach products such as peach juice (Wang et al., 2020), peach vinegar (Budak et al., 2021), and fruit wine have been prepared. Among these, fruit wine production is a suitable method for the deep processing of many fruits while still retaining the fruit’s flavor and some of its beneficial compounds.

Aroma is responsible for a fruit’s distinct flavor and has been extensively researched for its impact on consumer acceptability. Aroma is influenced by natural factors (e.g., fruit varieties and climatic conditions) (Sivilotti et al., 2017), winemaking techniques (Hu et al., 2018), and the presence of indigenous microorganisms (e.g., bacteria, yeasts, and filamentous fungi) (Liu et al., 2017). Several recent studies have focused on the changes in peach aroma throughout the ripening and storage stages (Bianchi et al., 2017), and the volatiles present in flat peach juice have also been explored (Zhang et al., 2010; Wang et al., 2019). More than 100 volatile compounds have been discovered across different peach cultivars, with esters, lactones, aldehydes, alcohols, and ketones being the most commonly found (Wang et al., 2009). Of these, lactones have the greatest effect on flat peach aroma (Sánchez et al., 2013). The aromatic active components in flat peach juice and their contribution have also been verified (Tan et al., 2022). The variability of esters is believed to underly the large differences in flavor quality between fresh flat peach juice and flat peach juice products (Wang et al., 2020).

In recent years, high-throughput sequencing (HTS) combined with multivariate data analysis has been widely used to identify microorganisms in many environments, such as the gut (Ross et al., 2019), as well as in food (Wang et al., 2018). This approach serves as a potent tool for studying the microbial diversity of fermented foods and the quality of fruit wines. The fermentation process of fruit wines is generally referred to as the fermentation process of wine. Usually, the microorganisms present in the skin of the fruit participate in the fermentation process of fruit wines together with the large variety of microorganisms present in the environment (picking, transporting, and crushing, etc.) (Chanprasartsuk et al., 2010). During the fermentation process, these bacteria generate a variety of metabolic substances that impact the flavor, safety, and product quality and stability of fruit wines (Xu X. et al., 2021). Flat peach wine fermentation can be classified into two types based on whether fermenters are used: inoculated fermentation and spontaneous fermentation. Spontaneous fermentation, caused by complex indigenous microorganisms, can provide more complex and richer wine flavors than inoculated fermentation (Lu et al., 2021). The native microorganisms present on the fruit skin serve as important microbial resources and contribute greatly to spontaneous fermentation (Raybaudi-Massilia et al., 2009). *Hanseniaspora uvarum*, *Issatchenkia terricola*, *Wickerhamomyces umomyces*, *Pichia kudriavzevii*, and *Lachancea thermotolerans* are among the microorganisms that have been discovered to contribute to the flavor of fruit wines (Belda et al., 2016; Shi et al., 2019). However, the succession pattern of microorganism populations during the spontaneous fermentation of flat peach wine and their corresponding metabolic characteristics have not been reported yet.

Spontaneously fermented flat peach wine is rich in flavor and aromatic compounds, but the microbiota involved in its fermentation remains unclear. In this study, HTS and headspace solid phase microextraction (HS-SPME) coupled with gas chromatography-mass spectrometry (HS-SPME-GC-MS) was used to examine microbial succession and flavor changes during the spontaneous fermentation of flat peach wine. Subsequently, multivariate data analysis was used to explore the characteristics of the spontaneous fermentation process of flat peach wine to provide a theoretical basis for the development of high-quality flat peach wine.

## Materials and Methods

### Spontaneous Fermentation and Sample Collection

Flat peaches [*Prunus persica* (L.) Batsch cv. “Yingger”] were picked in August 2021 from a flat peach orchard in Shihezi, Xinjiang Uygur Autonomous Region, China. The picked flat peaches were crushed into a homogenized pulp using a pulper. The biochemical composition of these flat peaches was then determined: the residual sugar content was 134 g/L; total acidity, 5.5 g/L; pH, 4.3; and soluble solids 14.6°Bx. Then, pectinase 30 mg/L was added, and the total sugar level was adjusted to 220 g/L. Fermentation was carried out in a 5 L fermenter at 16 ± 0.5°C under static fermentation conditions. Then, 150 mL of the fermentation liquid was collected on days 0, 3, 6, 9, 13, and 16 of fermentation (A, B, C, D, E, and F, respectively). All samples were centrifuged for 10 min at 4°C and 8,000 × *g*. The precipitate was collected for HTS, while the supernatant was used to analyze the volatile compounds (Jia et al., 2020).

### Determination of Physicochemical Properties

During fermentation, the pH, residual sugars, total acidity, and alcohol content were measured at points A–F. The residual sugar content was assessed using the dinitro salicylic acid method, and the pH was measured using a calibrated pH meter (Miller, 1959). The national standard GB/T 15038-2006 “General analytical procedure for wine and fruit wine” was used to detect total acid and alcohol content. The organic acids were examined using high-performance liquid chromatography (HPLC) using a modified version of a previously reported method (Ryan et al., 2020). Each sample was centrifuged and filtered into an injection vial using a 0.45μm filter. A Dikma C18 chromatographic column (5 m, 4.6 mm, 250 mm; Diamonsil Plus Technology, China) was then used. The mobile phase was a mixture of 0.1% phosphoric acid and methanol, the flow rate was 0.7 mL/min, and the column temperature was 40°C. UV detection was performed at 210 nm. Each indicator was assessed three times.

### Determination of Volatile Compounds

The volatile compounds in flat peach wine were detected using the method described by Tufariello et al. (2019), with some modifications. Each sample (10 mL) was placed in a 25-mL SPME glass vial along with 0.1 g/mL NaCl and 2 μL of the internal standard, 3-octanol (30 mg/mL). Subsequently, SPME fibers (DVB/CAR/PDMS 50/30 μm; Supelco, Bellefonte, PA, United States) were inserted into the glass vials and exposed to the headspace for 40 min at 40°C. Then, they were removed and inserted into the inlet of the GC column (HP INNOWAX column, 30 m × 0.25 mm; Agilent) for desorption for 7 min at 210°C. The inlet temperature was 230°C, the carrier gas was helium with a flow rate of 1 mL/min, and the electron energy was 70 eV.

The GC procedure was performed under the following conditions: 5 min at 40°C, temperature increase of 4°C/min until 86°C, 86°C for 5 min, temperature increase of 1.5°C/min until 90°C, temperature increase of 5°C/min until 180°C, 180°C for 3 min, temperature increase of 10°C/min until 230°C, and 230°C for 5 min. A computer search was used to match compounds to the NIST 14 Library. The assay’s accuracy was validated through comparisons with recognized compounds described in the literature, and the concentration of each constituent was measured using the internal standard 3-octanol.

### Microbial Diversity Analysis

The OMEGA Soil DNA Kit (M5635-02) was used to extract total genomic DNA samples (Omega Bio-Tek, Norcross, GA, United States). Spectrophotometry and agarose gel electrophoresis were used to determine the amount and quality of isolated DNA samples. The forward primer ITS5F (5′-GGAAGTAAAAGTCGTAACAAGG-3′) and the reverse primer ITS1R (5′-GCTGCGTTCTTCATCGATGC-3′) were used to amplify the fungal *ITS1* region using PCR. Moreover, the forward primer 338F (5′ACTCCTACGGGAGGCAGCA-3′) and reverse primer 806R (5′-GGACTACHVGGGTWTCTAAT-3′) were used for the PCR amplification of the V3–V4 region of the bacterial 16S rRNA gene. The cycle included initial denaturation at 98°C for 5 min; followed by 25 cycles of denaturation at 98°C for 30 s, annealing at 53°C for 30 s, and extension at 72°C for 45 s; and a final extension for 5 min at 72°C (Hao et al., 2016). QIIME2 2019.4 was used for microbiome bioinformatics, with minor modifications made according to protocols provided in the official tutorials^1^ (Bolyen et al., 2019).

### Statistical Analysis

In this study, three parallel tests were performed for each stage of the flat peach wine samples. Statistical analysis was performed using SPSS (version 20; IBM, Chicago, United States) software. Origin 2021 was used to generate histograms, and heat maps of volatile compounds were created using R (version 3.3.1). Multifactorial analysis was performed using Simca 14.1 software to analyze differences between microorganisms and volatile compounds, and the data were visualized using the Cytoscape (version 3.6.1) software.

## Results and Discussion

### Changes in Physicochemical Characteristics

#### General Physical and Chemical Indicators

**FIGURE 1:**
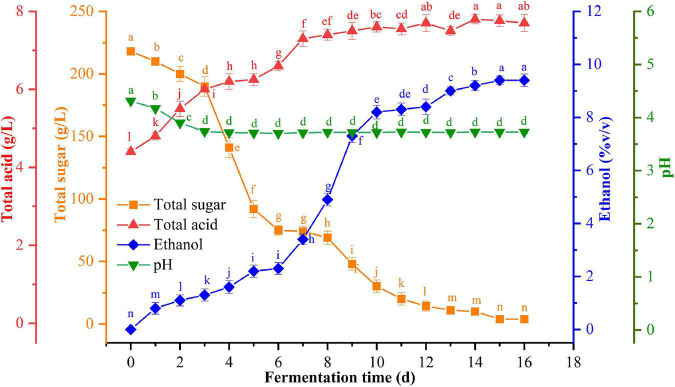
Changes in physicochemical indexes [total sugar (orange), ethanol (blue), total acid (red), and pH (green)] at different stages of fermentation.

The dynamic changes in the total sugars, pH, ethanol, and total acid content were detected at six time-points during the spontaneous fermentation of flat peach (Figure 1). During the fermentation process, the pH decreased from 4.3 to 3.7 and then remained relatively stable. The total acid concentration increased significantly from 4.4 g/L to 7.7 g/L (*p* < 0.05). The yeasts in the fermentation broth reproduced and grew by utilizing the fermentable sugars during the spontaneous fermentation of flat peach wine, causing the total sugar content to decline dramatically after the 3rd day. However, the total sugar content remained steady from the 16th day onward. Meanwhile, the yeast-mediated conversion of sugars to alcohol resulted in a significant increase in the ethanol concentration, which reached 9.4 ± 0.2% (v/v) on the 16th day and did not change thereafter. The gradual accumulation of ethanol inhibited the ability of the yeasts to metabolize and produce ethanol, consistent with prior findings (Chen et al., 2020).

#### Organic Acid Composition

**FIGURE 2:**
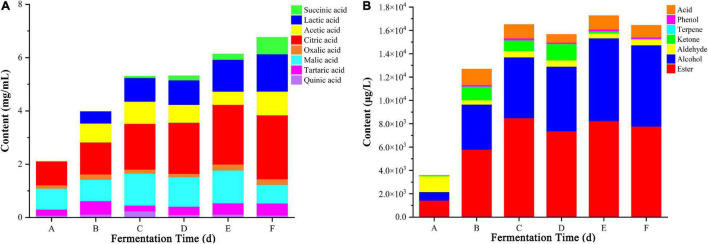
Changes in the levels of 8 organic acids **(A)** and 7 types of volatile substances **(B)** across different fermentation stages. A, B, C, D, E, and F Represent samples collected on fermentation days 0, 3, 6, 9, 12, and 16, respectively.

The flavor and taste of flat peach wine are strongly influenced by its organic acid concentration. In this study, eight organic acids (succinic, lactic, acetic, citric, oxalic, malic, tartaric, and quinic acid) were identified in the fermentation broth using HPLC (Figure 2A). The proportion of these organic acids varied with fermentation time. At the beginning of spontaneous fermentation, tartaric, malic, and citric acids were predominantly observed. However, with prolongation of the fermentation time, there was a continuous increase in the proportion of lactic acid, which was produced by bacteria such as *Leuconostoc* and *Lactobacillus*. There was also an overall trend for increasing levels of citric acid. In contrast, the levels of oxalic, tartaric, and malic acid remained relatively stable during the fermentation process. At the final stage, the organic acid composition of the flat peach wine was as follows: succinic acid (9.32%), lactic acid (20.71%), acetic acid (13.16%), citric acid (35.5%), oxalic acid (3.1%), malic acid (10.36%), tartaric acid (6.66%), and quinic acid (1.19%). Spontaneous fermented flat peach wine has a sour taste. This harmonious acidity is determined by the substrate’s composition ratio as well as the detectable concentration levels (Joshi et al., 2017). Considering the differences in acidity, in general, tartaric acid has a citrus-like flavor, malic acid has a metallic and green apple-like flavor, lactic acid has a tart and spicy taste, citric acid is fresh and pleasantly citrusy, and succinic acid has a sour, salty, and bitter taste. Each organic acid comparts different sensory characteristics to fruit wines (Mato et al., 2005; Izquierdo-Llopart et al., 2020).

### Microbial Succession and Interactions

#### Sequencing Quality Assessment

HTS was used to assess the microbiological diversity and communities present at six different time-points during the spontaneous fermentation of flat peach wine. For the bacterial communities, after removing low-quality sequences and chimeras, valid sequences were obtained for each sample. Accordingly, 688,716 high-quality sequences were obtained from all samples. For the fungal communities, 474,803 high-quality sequences were obtained. The number of bacterial sequences and operational taxonomic unites (OTUs) significantly outnumbered the number of fungal sequences and OTUs. For all samples, the coefficient curve sections were flat, and the coverage of high-quality sequences was greater than 99%, indicating that the sequencing results were adequate to indicate the microbiological variety of the samples (Supplementary Figures 1, 2). After standardization, the α-diversity of the samples was analyzed. The measures of species richness and diversity, such as Chao1 and Shannon index, are shown in Supplementary Table 1. The findings revealed that the quantity and diversity of bacterial and fungal communities changed during the fermentation of flat peach wine. The Chao1 index, the observed species, and the Shannon and Simpson indices of the fungal communities showed a decreasing trend, indicating that the diversity and richness of fungi gradually decreased during fermentation. In contrast, bacterial communities showed the opposite trend (Supplementary Figure 3). The common and unique bacterial OTUs observed at different stages of fermentation were also characterized. There were 164, 127, 138, 115, 60, and 66 fungal OTUs on days 0, 3, 6, 9, 13, and 16, respectively. Of these, 18 OTUs were common to all six fermentation stages. For bacteria, 162, 126, 145, 118, 114, and 109 OTUs were detected at different stages, respectively, with 49 OTUs common to all stages (Supplementary Figure 4). Thus, the microbial community structure in flat peach wine showed differences across different fermentation stages.

#### Microbial Succession

HTS was used to fully characterize the bacterial and fungal communities present during the spontaneous fermentation of flat peach wine and reveal the diversity and succession of microbial communities. Eighteen wine samples were collected from six fermentation processes, and the number of fungal and bacterial taxa at each classification level was examined during the fermentation of flat peach wine using Illumina sequencing (Supplementary Figure 5). The numbers of taxa detected at the genus level for fungi and bacteria were 48 and 40 at day 0, respectively. However, at day 16, the number of detectable taxa at the genus level gradually decreased to 20 and 30, respectively.

**FIGURE 3:**
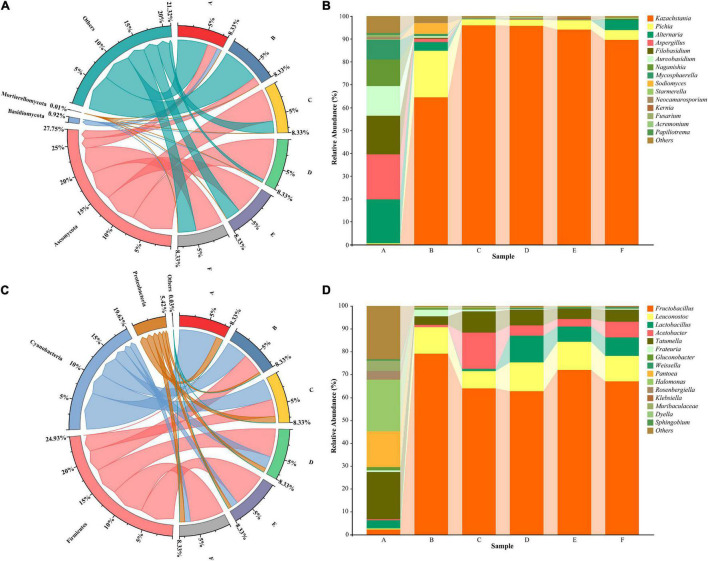
Differences in fungal **(A)** and bacterial **(C)** communities after different fermentation periods at the phylum level. The relative abundance of the top 15 fungi **(B)** and bacteria **(D)** at the genus level. A, B, C, D, E, and F Represent samples collected on fermentation days 0, 3, 6, 9, 12, and 16, respectively.

In order to assess community succession during fermentation, sequencing data were classified at the phylum and genus levels. The results showed that in the fungal community, the species distribution of the Ascomycota phylum was denser than that of other phyla, accounting for 27.75% of the total microbiota (Figure 3A). Ascomycetes were the dominant fungi during the fermentation process. In contrast, Firmicutes, Cyanobacteria, and Proteobacteria were the dominant bacterial phyla, accounting for 24.93, 19.62, and 5.42% of the bacteria, respectively (Figure 3C). These results were consistent with those from a previous study (Zhu et al., 2021). In the early phases of fermentation, Cyanobacteria and Anaplasma were predominant. However, their abundance declined with the growth of Firmicutes, which substantially increased in proportion after day 2.

At the genus level, the fungi *Aspergillus* (19.67%), *Alternaria* (19.06%), *Filobasidium* (16.97%), *Aureobasidium* (13.05%), *Naganishia* (11.53%), and *Mycosphaerella* (8.53%) had the highest relative abundance at stage A (Figure 3B). These findings differed from previous findings, likely owing to the cultivar of the flat peaches used and their origin. Notably, *Kazachstania* and *Pichia* gradually became dominant during the middle and late stages of fermentation. *Kazachstania*, a common yeast found in kimchi (Suzuki et al., 2018), sourdough (Minervini et al., 2015; Decimo et al., 2017), and feed (Santos et al., 2017), is strongly linked with the formation of flavor substances such as acids and alcohols in fermented products. *Pichia*, a fungal genus commonly found in fermented foods, can secrete esterases to promote the biocatalytic synthesis of ethyl ester compounds, which are important flavor substances in strong spiced white wine (van Rijswijck et al., 2019; Vicente et al., 2021). The relative abundance of *Aspergillus* showed significant fluctuations during fermentation, which may account for the increased alcohol concentration. Moreover, it has been reported that *Aspergillus* are the main microorganisms responsible for citric acid accumulation. This may be why flat peach wine has a higher citric acid content.

The bacterial diversity during flat peach wine fermentation was higher than the fungal diversity (Figure 3D). The main bacterial genera present at stage A of fermentation were *Tatumella* (20.57%), *Halomonas* (20.32%), and *Pantoea* (15.84%). The percentage of these three genera decreased as fermentation proceeded, probably due to the high enrichment of lactic acid bacteria in the later stages. *Pantoea* is an endophytic bacterium that is widely present on plant surfaces, grains, and fruits (Megías et al., 2016; Jung et al., 2018; Kour et al., 2021). The bacteria, detected at stage A, probably originated from the raw materials and environment. Subsequently, *Fructobacillus* maintained a relatively high abundance from stage B onward and was the dominant bacterial genus during flat peach wine fermentation. *Leuconostoc* and *Lactobacillu* showed a moderate relative abundance. These microorganisms typically grow better under low-oxygen and low-pH post-fermentation conditions as they are typically facultative anaerobes and acid-tolerant bacteria (McDonald et al., 1990). *Leuconostoc* can not only produce some flavor compounds, such as acetaldehyde and ethyl acetate, but can also secrete substances such as glucans (Yang X. et al., 2020). *Lactobacillus* is also one of the most significant genera involved in fruit wine production. It can create lactic acid and antimicrobial compounds such as bacteriocins, which limit the growth of pathogens and spoilage bacteria during the brewing process, increasing the flavor of fruit wine (Xu Z. et al., 2021). In addition to these bacteria, *Gluconobacter*, a functional bacterium, was also present in small amounts at stages A, B, and C. *Gluconobacter* is generally sensitive to alcohol, and its growth can be suppressed by high levels of alcohol (Habe et al., 2021). Hence, its abundance dropped significantly throughout the fermentation process in the present study. *Gluconobacter* is reported to be among the dominant flora in wine (Philippe et al., 2018) and white wine (Battling et al., 2020). Interestingly, pathogenic bacteria such as *Klebsiella* and *Rosenbergiella* were discovered during the fermentation process. However, as brewing progressed, their quantity reduced, possibly due to an increase in the abundance of lactic acid bacteria in the latter stages of fermentation.

Microbial diversity was assessed using principal component analysis (PCA) to determine the variations and similarity in microbial communities (Supplementary Figure 6). The PCA based β-diversity analysis of fungal and bacterial structures during wine fermentation showed that the first principal component (PC1) explained 50.2% of the variation between samples. Moreover, the second principal component (PC2) explained 16.4% of the variation. The contribution of PC1 to the bacterial structure was 46.8%, while the contribution of PC2 was 18.4%. As fermentation progressed, the microbial composition of the samples changed. The microbiota at stage A was different from that at other stages. However, the microbiota was comparable between stages E and F, toward the end of fermentation, because the samples were similar. In conclusion, the findings suggested that the size of the microbial population varies depending on the stage of fermentation.

#### Microbial Interrelationships

**FIGURE 4:**
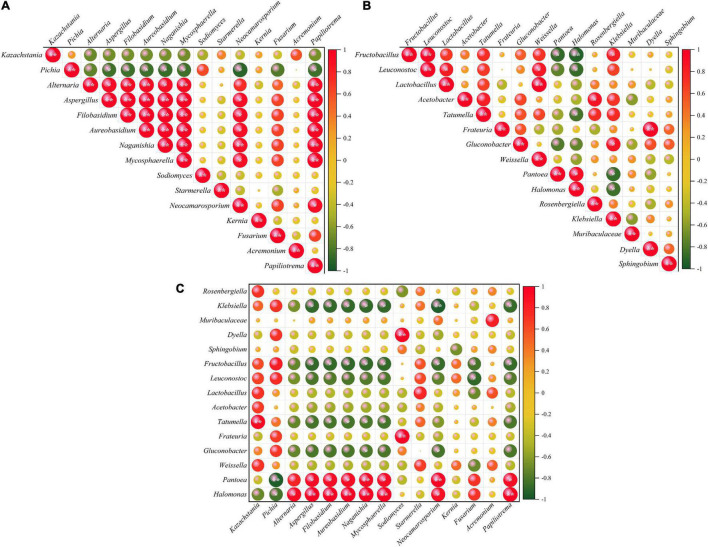
Co-occurrence and co-exclusion relationships between different bacteria **(A)** and fungi **(B)** and between bacteria and fungi **(C)**. The Pearson rank correlation matrix showing the abundance of the top 30 fungi and bacterial genera is depicted. Strong correlations are indicated by large circles, whereas weak correlations are indicated by small circles. The color of the scale bar denotes the nature of the correlation, with 1 indicating a perfect positive correlation (red) and -1 indicating a perfect negative correlation (green). Significant correlations (| *r*| > 0.7, *P* < 0.01) and (| *r*| > 0.9, *P* < 0.01) are indicated by * and **, respectively.

Microbial interactions are considered to be important for supporting the microbial community structure (Tempère et al., 2018). Pearson rank coefficients reveal whether microorganisms have positive or antagonistic associations. In this study, correlation analysis of fungal genera showed that *Pichia* was weakly negatively correlated with almost all other fungal genera except *Sodiomyces*, *Starmerella*, and *Kernia*. Further, similar findings were observed for *Kazachstania*. In contrast, *Alternaria*, *Filobasidium*, *Aureobasidium*, *Naganishia*, and *Mycosphaerella* showed a co-occurrence pattern with *Neocamarosporium* and *Papiliotrema* (Figure 4A). Among the bacteria, *Papiliotrema* and *Leuconostoc* showed negative correlations with *Pantoea* and *Halomonas*. *Fructobacillus*, *Lactobacillus*, and textitFrateuria were positively correlated with *Leuconostoc*, *Enterococcus*, and *Dyella*, respectively (Figure 4B). In addition, a correlation analysis of bacteria and fungi showed that *Pantoea* and *Halomonas* were also positively correlated with *Alternaria*, *Aspergillus*, *Filobasidium*, *Aureobasidium*, *Naganishia*, *Mycosphaerella*, *Neocamarosporium*, *Fusarium*, and *Papiliotrema.* In contrast, *Fructobacillus*, *Leuconostoc*, *Tatumella*, *Gluconobacter*, and *Klebsiella* showed exclusion patterns with *Alternaria*, *Aspergillus*, *Filobasidium*, *Aureobasidium*, *Naganishia*, and *Mycosphaerella* (Figure 4C).

The HTS results revealed that as the duration of fermentation and environment changed, the dominant bacterial genera in flat peach wine also changed. This implies that some microbial taxa, such as *Pantoea*, *Halomonas*, *Klebsiella*, *Aureobasidium*, and *Papiliotrema*, may be unable to adapt to the selective environment of accumulating ethanol levels and increasing acidity created by *Fructobacillus*, *Leuconostoc*, *Kazachstania*, and *Pichia*. In contrast to previous reports that *Lactobacillus* was the dominant flora in rice wine (Yang Y. et al., 2020) and koumiss (Meng et al., 2021), we found that *Fructobacillus* and *Leuconostoc* were the dominant lactic acid bacteria during the spontaneous fermentation flat peach wine and were also the probably main cause of pH fluctuations (Huang et al., 2018). During the brewing process, these bacteria can produce antimicrobial compounds such as bacteriocins that limit the growth of a vast number of microorganisms, including pathogens and spoilage germs (Collins et al., 2018). As dominant fungal genera, *Kazachstania* and *Pichia* exhibit efficient fermentation catabolism and tolerance to acid and ethanol and are highly competitive and dominant throughout the fermentation process. Some species of *Kazachstania*, a non-*Saccharomyces* yeast, have been shown to have a fermentation biology similar to that of brewer’s yeast (Merico et al., 2007).

### Changes in Volatile Compounds

#### Composition and Cluster Analysis of Volatile Compounds

The volatile compounds in flat peach wine were analyzed using HS-SPME-GC-MS. The changes in the composition and content of volatiles in flat peach wine during spontaneous fermentation are shown in Figure 2B. A total of 53 volatile compounds were detected at the six time-points (Table 1), including esters (23), alcohols (13), aldehydes (6), ketones (2), terpenes (3), phenol (1), and acids (5). The main constituents of flat peach are esters, aldehydes, and alcohols. The complexity of the volatiles increased with the duration of fermentation, especially for esters and alcohols. The esters that give flat peach wine its distinct flavor can be synthesized by esterifying alcohols with fatty acids. Moreover, microbial cells can generate these molecules using acetyl coenzyme A and higher alcohols as substrates during fermentation through alcohol acetyltransferases (Wang et al., 2014).

**TABLE 1:** Dynamic changes in the content of flavor compounds during the fermentation process (μg/L).

Compounds	RI	A	B	C	D	E	F	Odor threshold	OAV^1^
**Esters**									
Ethyl acetate	612	744.07 ± 0.58*^c^*	3216.51 ± 152.25*^a^*	1968.87 ± 117.83*^b^*	1576.59 ± 124.87*^c^*	1997.31 ± 191.85*^b^*	3170.56 ± 324.2*^a^*	7750	0.1–1
Isoamyl acetate	876	Nd	145.62 ± 3.83*^b^*	296.36 ± 0.61*^a^*	142.94 ± 9.15*^b^*	158.39 ± 5.93*^b^*	19.14 ± 2.20*^c^*	30	0.1–1
Pentyl acetate	911	Nd	Nd	Nd	Nd	Nd	12.09 ± 0.77*^a^*	43	0.1–1
Ethyl hexanoate	1,000	Nd	31.42 ± 0.83*^e^*	133.51 ± 2.34*^ab^*	114.04 ± 2.86*^bc^*	160.72 ± 6.87*^a^*	71.25 ± 0.88*^d^*	5	> 1
Hexyl acetate	1,011	44.12 ± 0.63*^d^*	995.05 ± 3.13*^a^*	635.89 ± 25.22*^b^*	318.01 ± 12.73*^c^*	238.37 ± 5.92*^c^*	60.26 ± 0.89*^d^*	670	< 0.1
3-Hexen-1-yl acetate	1,005	132.04 ± 22.66*^a^*	21.67 ± 0.72*^c^*	10.14 ± 0.01*^c^*	120.56 ± 6.05*^a^*	96.22 ± 0.16*^b^*	24.37 ± 0.26*^c^*	13	> 1
Ethyl lactate	815	Nd	Nd	176.19 ± 0.13*^c^*	256.21 ± 2.27*^b^*	397.98 ± 4.23*^a^*	262.76 ± 9.02*^b^*	154,636	< 0.1
Isoamyl lactate	1,047	Nd	1.37 ± 0.04*^c^*	12.17 ± 0.83*^b^*	15.51 ± 0.2*^b^*	24.68 ± 1.9*^a^*	21.05 ± 1.45*^a^*	/	
3-Hexenyl isobutyrate	1,145	13.69 ± 1.53*^a^*	0 ± 0	Nd	Nd	Nd	Nd	/	
2-Hexen-1-ol acetate	1,006	105.4 ± 0.78*^a^*	56.21 ± 4.23*^b^*	26.86 ± 0.45*^c^*	3.31 ± 0.22*^d^*	Nd	Nd	/	
Ethyl caprylate	1,196	8.97 ± 0.26*^d^*	24.86 ± 0.26*^c^*	235.17 ± 3.66*^a^*	33.3 ± 2.33*^c^*	245.67 ± 4.01*^a^*	190.71 ± 7.57*^b^*	5	> 1
Ethyl non-anoate	1,296	3.41 ± 0.15*^d^*	1.69 ± 0.31*^d^*	40.99 ± 0.7*^b^*	138.45 ± 2.44*^a^*	29.94 ± 1.37*^c^*	29.09 ± 2.89*^c^*	1,300	< 0.1
Ethyl caprate	1,396	2.74 ± 0.01*^d^*	53.55 ± 0*^c^*	355.53 ± 10.98*^a^*	10.02 ± 0.01*^d^*	357.58 ± 5.36*^a^*	225.95 ± 18.35*^b^*	200	> 1
Ethyl benzoate	1,171	11.63 ± 0.47*^c^*	23.99 ± 2.57*^b^*	15.4 ± 0.28*^c^*	15.07 ± 0.05*^c^*	20.08 ± 0.76*^b^*	934.12 ± 24.13*^a^*	53	> 1
Ethyl phenylacetate	1,246	Nd	3.17 ± 0.05*^c^*	2.67 ± 0.12*^c^*	4.57 ± 0.4*^b^*	6.97 ± 0.69*^a^*	Nd	155.5	< 0.1
Ethyl laurate	1,595	Nd	Nd	15.1 ± 0.07*^c^*	46.96 ± 4.92*^a^*	49.88 ± 1.33*^a^*	26.33 ± 0.94*^b^*	500	< 0.1
Phenethyl acetate	1,258	329.44 ± 2.99*^d^*	752.9 ± 2.33*^c^*	3985.34 ± 272.48*^a^*	3505.94 ± 4.2*^a^*	3406.42 ± 66.17*^a^*	2514.08 ± 9.96*^b^*	1,800	> 1
Ethyl tetradecanoate	1,794	Nd	Nd	1.23 ± 0.01*^c^*	2.77 ± 0.54*^b^*	2.43 ± 0.02*^b^*	7.44 ± 0.31*^a^*	2,000	< 0.1
γ-Decalactone	1,470	434.39 ± 45.81*^a^*	375.85 ± 30.84*^b^*	152.88 ± 2.04*^c^*	84.94 ± 3.49*^d^*	154.7 ± 3.32*^b^*	110.02 ± 7.09*^c^*	1.1	> 1
Ethyl palmitate	1,993	Nd	Nd	2.71 ± 0.5*^c^*	15.39 ± 2.4*^a^*	8.05 ± 0.04*^b^*	17.06 ± 0.75*^a^*	1,000	< 0.1
Tetradecalactone	1,935	Nd	Nd	Nd	Nd	17.73 ± 0.23^ a^	13.28 ± 0.15*^b^*	29	0.1–1
γ-Dodecalactone	1,678	10.09 ± 1.62*^a^*	4.02 ± 0.85*^c^*	4.45 ± 0.32*^c^*	4.27 ± 0.19*^c^*	9.17 ± 0.04*^a^*	6.09 ± 0.06*^b^*	0.43	> 1
Methyl benzoate	1,094	6.67 ± 0.08*^d^*	40.12 ± 0.01*^c^*	367.72 ± 5.46*^b^*	502.98 ± 9.18*^b^*	823.4 ± 16.55*^a^*	20.58 ± 1.82*^c^*	73	0.1–1
**Alcohols**									
Ethanol	324	Nd	986.83 ± 47.26*^c^*	2398.01 ± 140.01*^a^*	2196.84 ± 139.8*^a^*	886.33 ± 61.04*^b^*	233 ± 8.49*^d^*	950	> 1
3-Methyl-1-butanol	736	Nd	126.23 ± 18.55*^d^*	791.3 ± 7.99*^c^*	1111.01 ± 78.5*^b^*	1309.73 ± 77.59*^a^*	874.02 ± 2.84*^c^*	7,000	0.1–1
1-Hexanol	868	Nd	1221.71 ± 1.21*^a^*	830.23 ± 14.3*^b^*	731.61 ± 22.35*^c^*	843.07 ± 4.9*^b^*	533.48 ± 9.53*^d^*	5,200	0.1–1
3-Hexen-1-ol	852	186.32 ± 21.46*^a^*	162.09 ± 8.55*^a^*	105.73 ± 4.76*^c^*	85.51 ± 0.36*^d^*	112.82 ± 1.99*^b^*	96.68 ± 4.72*^d^*	400	0.1–1
Cyclohexanol	880	576.53 ± 0.12*^a^*	356.79 ± 26.01*^b^*	Nd	Nd	Nd	Nd	300	
2-Octanol	998	Nd	Nd	Nd	5.93 ± 0.66*^a^*	4.8 ± 0.57*^b^*	Nd	120	
Cyclohexanemethanol	/	103.76 ± 2.48*^a^*	Nd	Nd	Nd	Nd	Nd	/	
1-Octanol	1,071	Nd	18.98 ± 1.36*^a^*	14.96 ± 2.13*^b^*	4.26 ± 0.12*^c^*	21.37 ± 2.87*^a^*	Nd	800	
2,3-Butanediol	1,071	Nd	18.98 ± 2.81*^ab^*	14.96 ± 0.47*^b^*	4.26 ± 0.54*^c^*	21.37 ± 2.38*^a^*	Nd	800	
2-Non-anol	/	Nd	2.7 ± 0.49*^d^*	8.9 ± 0.64*^c^*	83.75 ± 2.65*^b^*	15.79 ± 0.56*^c^*	143.46 ± 9.52*^a^*	20–50	> 1
Benzyl alcohol	1,173	Nd	68.24 ± 12.9*^a^*	Nd	Nd	Nd	Nd	600	
Phenylethyl alcohol	1,036	12.29 ± 0.03*^d^*	584.32 ± 59.62*^a^*	422.52 ± 1.78*^b^*	284.16 ± 17.08*^c^*	506.31 ± 11.53*^a^*	287.62 ± 5.39*^c^*	2,0000	< 0.1
β-ionol	1,116	8.7 ± 0.3*^e^*	170.91 ± 43.07*^d^*	583.62 ± 2.56*^c^*	492.85 ± 9.09*^c^*	1061.17 ± 43.96*^a^*	869 ± 48.79*^b^*	10,000	< 0.1
**Aldehydes**									
Hexanal	800	221.18 ± 0.69*^a^*	5.03 ± 1.44*^b^*	Nd	Nd	Nd	Nd	5–15	
2-Hexenal	851	1070.62 ± 3.83*^a^*	Nd	Nd	Nd	Nd	Nd	30	
2,4-Heptadienal	1,012	2.91 ± 0.29*^a^*	Nd	Nd	Nd	Nd	Nd	15.4	
Benzaldehyde	962	4.23 ± 0.02*^d^*	256.52 ± 18.75*^b^*	384.66 ± 21.68*^a^*	238.98 ± 6.35*^b^*	164.84 ± 3.42*^c^*	133.46 ± 2.45*^c^*	2,000	< 0.1
2,5-Dimethylbenzaldehyde	1,208	56.73 ± 0.34*^d^*	85.65 ± 4*^d^*	135.94 ± 11.27*^c^*	282.24 ± 15.73*^b^*	245.35 ± 0.25*^b^*	347.97 ± 5.64*^a^*	200	> 1
4-Undecanolide	1,576	Nd	Nd	2.03 ± 0.02*^a^*	1.98 ± 0.34*^b^*	1.87 ± 0.01*^b^*	Nd	2.1	0.1–1
**Ketones**									
3-Octanone	986	10.85 ± 0.48*^a^*	10.87 ± 0.62*^a^*	4.56 ± 0.4*^b^*	1.79 ± 0.56*^c^*	Nd	Nd	21.4	
Geranylacetone	1,453	Nd	Nd	Nd	Nd	8.4 ± 0*^b^*	9.83 ± 0.59*^a^*	60	0.1–1
**Terpenes**									
Linalool	1,099	22.68 ± 0.02*^d^*	45.21 ± 0.15*^c^*	72.71 ± 8.99*^b^*	67.71 ± 1.92*^b^*	113.95 ± 10.57*^a^*	Nd	25	
β-Ionone	1,491	3.93 ± 0.9*^d^*	18.62 ± 0.44*^c^*	19.23 ± 0.16*^bc^*	20.23 ± 1.58*^b^*	23.54 ± 0.38*^ab^*	25.28 ± 4.44*^a^*	8.4	> 1
Geraniol	1,255	Nd	Nd	Nd	6.32 ± 0.23*^c^*	16.28 ± 0.91*^a^*	8.09 ± 0.06*^b^*	0.99	> 1
**Phenols**									
Eugenol	1,357	0.28 ± 0.01*^d^*	32.7 ± 1.91*^c^*	152.03 ± 29.72*^a^*	111.6 ± 1.13*^b^*	125.2 ± 10.75*^ab^*	136.87 ± 19*^a^*	5	> 1
**Acids**									
1-Hexanoic acid	990	10.11 ± 0.79*^d^*	463.64 ± 45*^a^*	122.07 ± 14.9*^b^*	55.1 ± 3.61*^c^*	112.97 ± 9.17*^b^*	66.41 ± 4.53*^c^*	420	0.1–1
Octanoic acid	1,180	1.3 ± 0.05*^d^*	62.56 ± 0.4*^b^*	75.29 ± 1.92*^b^*	43.78 ± 1.26*^c^*	116.29 ± 4.45*^a^*	41.37 ± 0.97*^c^*	500	< 0.1
Non-anoic acid	1,273	Nd	Nd	5.09 ± 0.06*^b^*	2.97 ± 0.69*^c^*	12.89 ± 2.04*^a^*	6.47 ± 0.05*^b^*	500–800	< 0.1
Decanoic acid	1,373	Nd	13.36 ± 0.96*^c^*	22.6 ± 1.84*^b^*	23.42 ± 2.42*^b^*	50.69 ± 7.56*^a^*	15.52 ± 1.07*^c^*	1,000	< 0.1
Benzoic acid	1,170	Nd	Nd	15.2 ± 0.14*^c^*	304.25 ± 31.89*^b^*	826.66 ± 54.21*^a^*	Nd	1,000	

*A, B, C, D, E, and F represent samples collected on fermentation days 0, 3, 6, 9, 12, and 16, respectively. Data are expressed as the means ± standard (n = 3). The different lowercase letters in each row indicate a significant difference between the samples (P < 0.05). Nd, not detected. RI, retention index; OAV, odor activity value.*

*^1^OAV was calculated by dividing concentration by the odor threshold value of the compound.*

**FIGURE 5:**
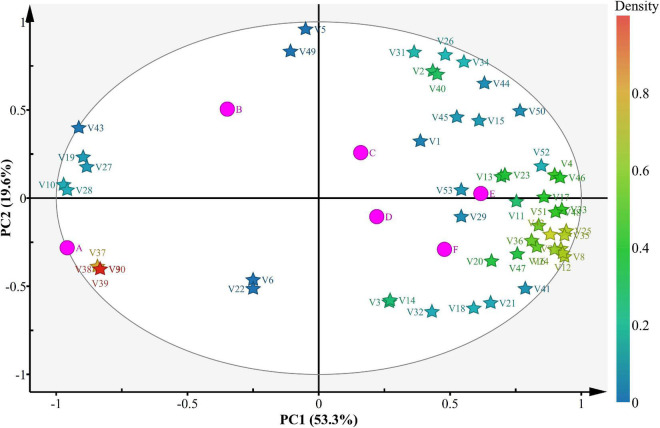
Bioplot of the PCA for volatile compounds present in the samples. A, B, C, D, E, and F Represent samples collected on fermentation days 0, 3, 6, 9, 12, and 16, respectively. “V” represents volatile compounds.

PCA was used to understand the relationships and differences between scent components in different wines (Figure 5). Among them, 53 different components explained 74.1% of the variance, with PC1 and PC2 explaining 53.3 and 19.6% of the variance, respectively. The volatile components at stage A were significantly different from those at stages B–F, indicating that the volatile components in flat peach wine were significantly different from those in the fermentation products generation during fermentation. Most volatile compounds were found at stages E and F, confirming the flat peach wine has a greater concentration of volatile components. The remaining samples contained only a few volatile compounds, consistent with the lower volatile compound concentrations observed in these samples throughout the experiment. These variations suggest that the aroma of the flat peach wine varied across different stages of fermentation. It is worth noting that at stage E, half of the volatile chemicals (alcohols and esters) were found in the lower right quadrant.

#### Characteristic Flavor Substances of Flat Peach Wine

**FIGURE 6:**
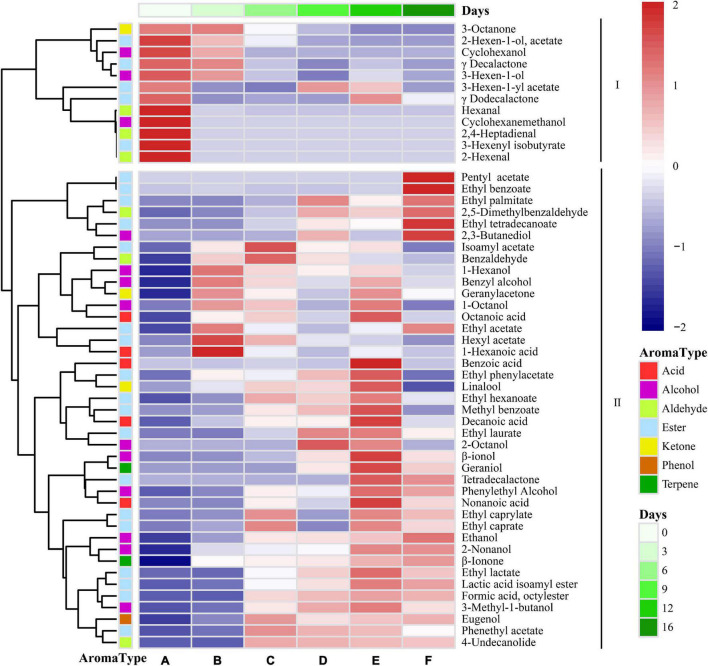
Heatmap cluster analysis of the volatile components identified during fermentation. The colors correspond to normalized mean levels, from low (blue) to high (red); the scale is shown at the top. A, B, C, D, E, and F) Represent samples collected on fermentations days 0, 3, 6, 9, 12, and 16, respectively.

The fragrance composition was studied during the spontaneous fermentation process, where the color intensity was related to the relative quantity of volatile chemicals, to better understand the dynamics of these volatile compounds (Figure 6). The volatile components could be split into two groups based on their patterns during the fermentation process. There were 12 major volatile compounds present in flat peaches, including 3-hexen-1-yl acetate, 3-octanone, hexanal, 3-hexen-1-ol, 2-hexenal, γ-decalactone, and δ-decalactone. This was consistent with previous findings (Zhu and Xiao, 2019). Hexanal, 3-hexen-1-ol, and 2-hexenal are C_6_ compounds that are generated as byproducts after the enzyme-catalyzed degradation of unsaturated fatty acids. Lactones, and particularly γ-decalactone and δ-decalactone, are the “character shock” components of flat peach flavor. There were 41 volatile compounds present in Class II, and these represented the main volatile substances produced during the late stage of spontaneous flat peach wine fermentation. Because these were present at greater quantities during the fermentation process, several of these volatile chemicals were intermediate products.

Esters are important flavor substances during the fermentation of flat peach wine because they confer fruit-like flavors (Tufariello et al., 2012). During the spontaneous fermentation of flat peach wine, the main esters were isoamyl acetate, pentyl acetate, ethyl laurate, ethyl caprate, ethyl acetate, hexyl acetate, phenethyl acetate, and ethyl caprylate. Due to the high concentration of alcohol and the corresponding presence of acetyl coenzyme A and acyltransferase, large amounts of acetate and ethyl esters were produced (Mason and Dufour, 2000; van Rijswijck et al., 2019). The main esters with odor activity values (OAV) > 1 at the end of fermentation were ethyl hexanoate (pineapple and banana aroma), 3-hexen-1-yl acetate (banana aroma), ethyl caprylate (fruit and wax aroma), ethyl caprate (fruit aroma), ethyl benzoate (fruity and medicinal fragrance), phenethyl acetate (fruity and floral fragrance), γ-decalactone (peach fragrance), and γ-dodecalactone. This could be due to the high acyltransferase and alcohol acetyltransferase expression during spontaneous fermentation (Procopio et al., 2011). Ethyl lactate and isoamyl acetate were identified during the middle and late phases of fermentation, indicating that they were mostly generated by yeast metabolism and esterification processes. In addition, during the mid-late fermentation stage, the concentration of phenethyl acetate—which is produced by yeasts from higher alcohols during fermentation and boosts olfactory complexity by providing banana, pear, and apple scents—rose significantly.

Alcohols are an important source of alcoholic sweetness and act as aroma enhancers for wine. They are precursors to esters, which provide floral and fruity aroma (Gao et al., 2014). The flat peach wine produced after spontaneous fermentation contained high concentrations of ethanol, 3-methyl-1-butanol, 1-hexanol, and benzyl alcohol. However, due to their relatively high odor threshold, these alcohols (with the exception of ethanol) did not favorably enhance the flavor of the wine. The concentration of these compounds decreased during fermentation, likely because they were used in esterification for the synthesis of the corresponding esters (Abeijón Mukdsi et al., 2018). Aldehydes and ketones are also important components of the aroma of fruit wines (Tufariello et al., 2012). Decanal, which provides fatty aromas and fatty flavors, had an OAV > 1 in flat peach wine. The OAV of benzaldehyde, a versatile aromatic aldehyde, was < 0.1 at the end of fermentation. The release of acid and the breakdown of flavor substances result in the formation of ketones through β-oxidation (Montel et al., 2014). Geranylacetone was present in trace amounts at the end of fermentation.

### Correlation Analysis Between Core Microbiota and Volatile Compounds

#### Screening of Core Microorganisms and Core Flavor Substances

Three criteria were used to identify the major functioning microorganisms during the fermentation process: (i) The abundance of the microorganisms and volatile compounds varied relatively steadily throughout the fermentation process; (ii) the significance of the predictive component (VIP) values for the microorganisms and volatiles was > 1.0; and (iii) the absolute value of the linear correlation coefficient between the distribution of volatile component concentrations and the distribution of relative microbe abundance was > 0.6 (Zheng et al., 2018).

Microorganisms (fungi and bacteria) with the 40 highest relative abundance levels during the spontaneous fermentation of flat peach wine were selected to identify the core microbiota. Variables (x) and attribute variables (y) were assigned to microorganisms and all volatile chemicals, respectively. VIP values were calculated for each fungal and bacterial group at the genus level with respect to the volatile compounds. VIP values > 1 for the fungal and bacterial groups are indicated in red and blue red, respectively, to indicate that the fungal and bacterial groups are important in the synthesis of volatile chemicals (Supplementary Figure 7). The VIP values of the analyzed microbes varied between 0.289 and 1.285; 25 microbial genera (VIP > 1.0), including 14 fungi and 11 bacteria, had a significant impact on volatile flavors. The principal contributors to the generation of volatile metabolites during the spontaneous fermentation of flat peach wine included *Lactobacillus*, *Caulobacter*, *Enterococcus*, *Fructobacillus*, *Acetobacter*, *Leuconostoc*, *Kazachstania*, *Pichia*, *Aspergillus*, *Vishniacozyma*, and *Naganishia*. Based on the three criteria listed previously, 27 core volatiles were chosen, including ethanol, benzyl alcohol, and eugenol (Supplementary Figure 8).

#### Microbe and Flavor Correlation Analysis

**FIGURE 7:**
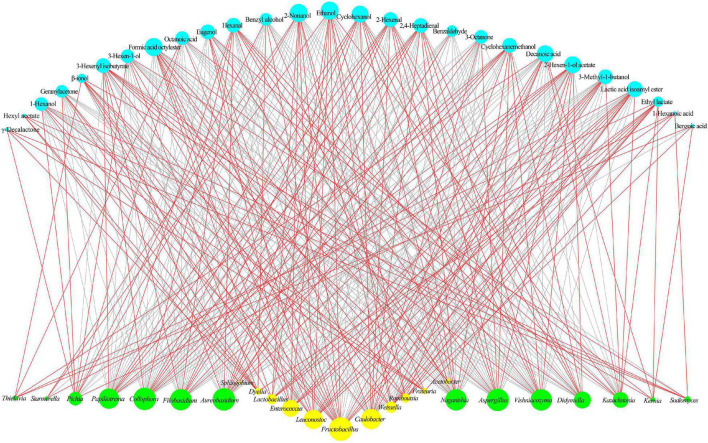
Correlation analyses between the microbiota and volatile compounds during fermentation. The core fungal and bacterial genera are shown in yellow and green, respectively. The core volatile compounds are shown in blue. Red and gray lines indicate positive and negative correlations, respectively.

During the spontaneous fermentation of flat peach wine, the correlation between the core microbiota and the core volatile compounds was investigated (Figure 7). Some alcohols and esters are favorably associated with yeasts typically found in fruit wines, such as *Kazachstania* and *Pichia*. *Kazachstania* is a non-*Saccharomyces* yeast that can assimilate lactic acid and hydrolyze glucuronide to provide metabolic substrates for heterofermentative lactic acid bacteria, enabling them to produce acetic acid from fructose (Corsetti et al., 2001). *Kazachstania* has also been shown to exhibit some probiotic effects (Kim et al., 2019). *Pichia* is an important producer of several secondary metabolites (Vicente et al., 2021). In this study, *Aspergillus*, *Filobasidium*, *Aureobasidium*, *Naganishia*, *Papiliotrema*, *Didymella*, *Collophora*, and *Vishniacozyma* were found to be highly correlated with aldehydes, alcohols, and esters (e.g., hexanal, cyclohexanol, and 3-hexenyl isobutyrate). Among them, *Aspergillus* shows a high level of environmental flexibility and is acid- and ethanol-resistant. *Aspergillus* has been used as a fermenting agent in some fermented foods and shown good results. *Vishniacozyma* also deserves attention as the predominant dominant fungi found in the grapes of organic vineyards in Xinjiang (Zhu et al., 2021) and in the ice wine produced in Yili, Xinjiang, China (Chen et al., 2020). Although *Vishniacozyma* was not the predominant dominant flora during the fermentation of flat peach wine, its contribution to volatile compounds cannot be ignored. According to Gramisci et al. (2018), *Vishniacozyma* produces antimicrobial compounds and enzymes to preserve its ecological niche, which may be connected to the generation of aldehydes and acids during fermentation.

Among the bacteria, the lactic acid bacteria *Fructobacillus*, *Leuconostoc*, *Lactobacillus*, and *Weissella* were associated with the production of 2-nonanol, ethanol, 3-methyl-1-butanol, octyl formate, isoamyl lactate, and ethyl lactate. According to reports, these lactic acid bacteria play a crucial role in fermented foods by delivering pleasant sensory and nutritional advantages (Kavitake et al., 2018). During the fermentation of foods, *Weissella* produces esters, organic acids, and short-chain fatty acids (Kamboj et al., 2015). This indicates that during the spontaneous fermentation of flat peach wine, *Weissella* could increase the development of taste compounds. Sumby et al. (2019) noted that *Leuconostoc* was positively correlated with ester production, and *Acetobacter* was positively correlated with organic acids and esters. This may be due to the enzymatic activity of these microbes, since *Acetobacter* synthesizes ethanol dehydrogenase and oxygenase, which stimulate the synthesis of acetic acid and competitively inhibit the enzymes involved in acetic acid catabolism (Trèek et al., 2015). *Sphingobium* was only found to show a high correlation with benzoic acid and β-ionol. Early reports suggest that *Sphingobium* can assimilate a large amount of carbon sources, reducing the level of volatile compounds (Poroyko et al., 2011).

This study shows that native microorganisms present on peach skin reflect the health of the peach and play an important role in the flavor and quality of flat peach wine. To our knowledge, this study is the first to use the HTS approach to evaluate microbial succession during the spontaneous fermentation of flat peach wine. The results of our multivariate analysis revealed a substantial association between bacteria and volatile compounds. However, further investigation using a multi-omics approach will be required in order to verify the relationships between core microorganisms and specific flavors. Moreover, additional studies on the locally dominant flora detected in this study may help elucidate their contribution to the sensory quality of flat peach wine, as well as microbiological safety.

## Conclusion

In this study, we monitored the dynamic changes in the physicochemical properties, volatile metabolite content, and microbial community succession during the spontaneous fermentation of flat peach wine. Spontaneous fermentation enhances the aromatic characteristics of flat peach wine. HTS revealed a significant decrease in fungal community diversity and a significant increase in bacterial community diversity with an increase in the duration of flat peach wine fermentation. An evaluation of the interactions between volatile components and the core functional microbiota at various fermentation stages revealed that microorganisms are important components of flat peach wine and are responsible for the generation of aromatic and volatile compounds (including many esters and alcohols) that are characteristic of flat peach wine. *Fructobacillus*, *Leuconostoc*, *Lactobacillus*, *Acetobacter*, *Kazachstania*, *Pichia*, and *Aspergillus* are the dominant microbes that contribute to microbial community succession and may also be related to the taste and flavor structure of flat peach wine. The study’s findings contribute to an improved understanding of the mechanism underlying the spontaneous fermentation of flat peach wine and lay the foundation for improved quality control during fermentation.

## Data Availability Statement

The data presented in the study are deposited in the NCBI repository. Available at: https://www.ncbi.nlm.nih.gov/sra, accession number PRJNA849518 and https://www.ncbi.nlm.nih.gov/biosample, accession numbers, 29093450–29093467.

## Author Contributions

XX performed the experiments, collected the test data, and drafted the manuscript. YM processed the data. HW and TL provided data for the study. PY, CL, and RZ made the graphs and tables. XS and BW designed the study and revised the manuscript. All authors reviewed the manuscript.

## Conflict of Interest

The authors declare that the research was conducted in the absence of any commercial or financial relationships that could be construed as a potential conflict of interest.

## Publisher’s Note

All claims expressed in this article are solely those of the authors and do not necessarily represent those of their affiliated organizations, or those of the publisher, the editors and the reviewers. Any product that may be evaluated in this article, or claim that may be made by its manufacturer, is not guaranteed or endorsed by the publisher.

## Footnotes

1
https://docs.qiime2.org/2019.4/tutorials/
