# Supplementary material for: A Snapshot of Microbial Succession and Volatile Compound Dynamics in Flat Peach Wine During Spontaneous Fermentation
Source: Front Microbiol. 2022 Jun 29;13:919047. doi: 10.3389/fmicb.2022.919047 (PMC9277550; doi:10.3389/fmicb.2022.919047)
Supplement: Supplementary file 1 [file Data_Sheet_1.docx]

Supplementary materials for:

**A snapshot of microbial succession and volatile compound dynamics in flat peach wine during spontaneous fermentation**

Xiaoyu Xu^1^, Yuanyuan Miao^1^, Huan Wang^1^, Piping Ye^1^, Tian Li^1^, Chunyan Li^1^, Ruirui Zhao^1^, Bin Wang^1^*, Xuewei Shi^1^*

^1^ Food college, Shihezi University, Shihezi 832000, Xinjiang Uygur Autonomous Region, P. R. China

* Corresponding authors

E-mail addresses: B. W.: binwang0228@shzu.edu.cn; X. S.: shixuewei@shzu.edu.cn

Tel.: 86-0993-2058093

Catalog

[Supplementary tables 1](#_Toc100523439)

[Table S1. Richness and diversity indexes of fungal communities in the flat peach wine. 1](#_Toc100523440)

[Supplementary figures 1](#_Toc100523441)

Figure S1. Assessment of sparse curves for fungal sequencing saturation…………………………1

[Figure S2. Assessment of sparse curves for bacterial sequencing saturation. 2](#_Toc100523442)

[Figure S3. Alpha diversity indexes (Chao 1 and Shannon) for fungal (A) and bacterial communities (B). 3](#_Toc100523443)

[Figure S4. Venn diagram of fungal (A) and bacterial (B) OTUs among samples. 4](#_Toc100523444)

[Figure S5. Statistics of the microbiota at each classification level in fungi (A) and bacteria (B). 5](#_Toc100523445)

[Figure S6. Principal component analysis (PCA) scatter plot of fungal (A) and bacterial communities (B) in the samples. 6](#_Toc100523446)

[Figure S7. Variable importance of predictive components (VIP [pred]) plot for the microbiota. The red, blue, and green columns refer to fungi (VIP [pred] > 1), bacteria (VIP [pred] > 1), and microbes (VIP [pred] < 1), respectively. 7](#_Toc100523447)

[Figure S8. Variable importance of predictive components (VIP [pred]) plot for volatile compounds. 7](#_Toc100523448)

# Supplementary tables

## Table S1. Richness and diversity indexes of fungal and bacterial communities in the flat peach wine.

| Samples | Fungi | | | | | Bacterial | | | |
| --- | --- | --- | --- | --- | --- | --- | --- | --- | --- |
|  | Chao1 | Observed species | Shannon | Simpson | Chao1 | | Observed species | Shannon | Simpson |
| A | 124.04±13.25^a^ | 123.23±15.44^a^ | 3.57±0.07^a^ | 0.85±0.15^a^ | 154.96±15.23^c^ | | 140.76±16.22^c^ | 0.82±0.03^f^ | 0.25±0.02^d^ |
| B | 109.80±10.20^b^ | 104.96±16.01^b^ | 3.11±0.02^b^ | 0.81±0.16^a^ | 187.46±15.32^c^ | | 174.86±13.65^c^ | 1.98±0.12^e^ | 0.54±0.11^c^ |
| C | 113.71±11.23^b^ | 105.20±12.35^b^ | 2.15±0.03^d^ | 0.65±0.15^b^ | 231.34±16.23^b^ | | 212.13±17.62^b^ | 2.23±0.15^d^ | 0.62±0.13^b^ |
| D | 94.92±12.2^c^ | 89.90±9.56^c^ | 1.69±0.01^e^ | 0.58±0.20^c^ | 246.08±15.23^a^ | | 229.23±14.85^a^ | 2.98±0.23^a^ | 0.72±0.09^a^ |
| E | 65.96±12.6^d^ | 63.63±7.23^d^ | 2.10±0.02^d^ | 0.65±0.23^b^ | 236.25±14.20^b^ | | 220.23±15.63^b^ | 2.59±0.25^c^ | 0.58±0.08^c^ |
| F | 61.35±6.5^d^ | 59.60±6.23^d^ | 1.68±0.01^c^ | 0.56±0.09^a^ | 232.30±15.20^b^ | | 216.43±18.63^b^ | 2.79±0.26^b^ | 0.62±0.11^b^ |

# Supplementary figures

##
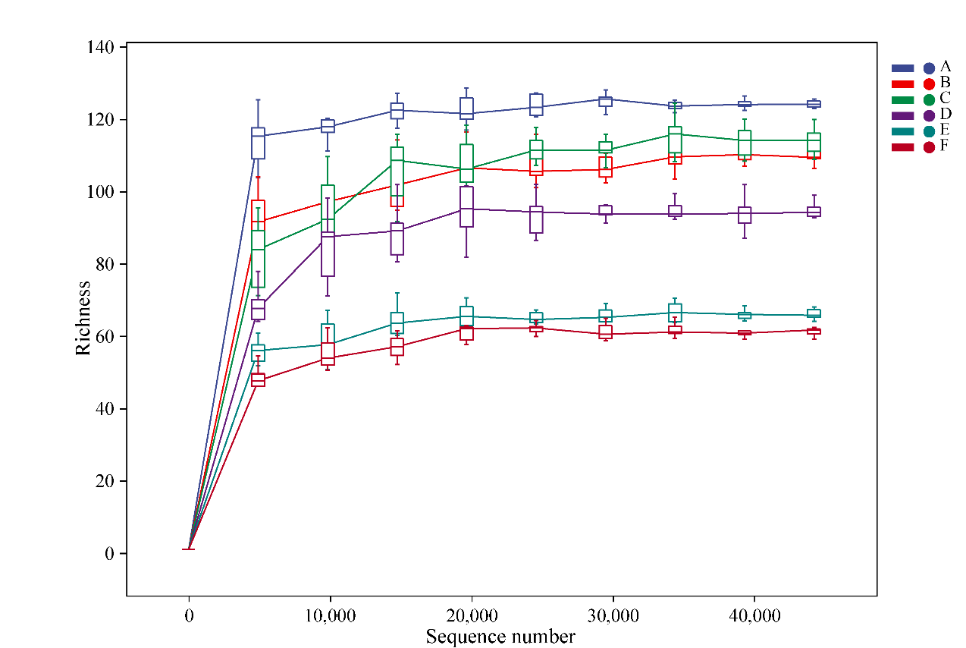
Figure S1. Assessment of sparse curves for fungal sequencing saturation.


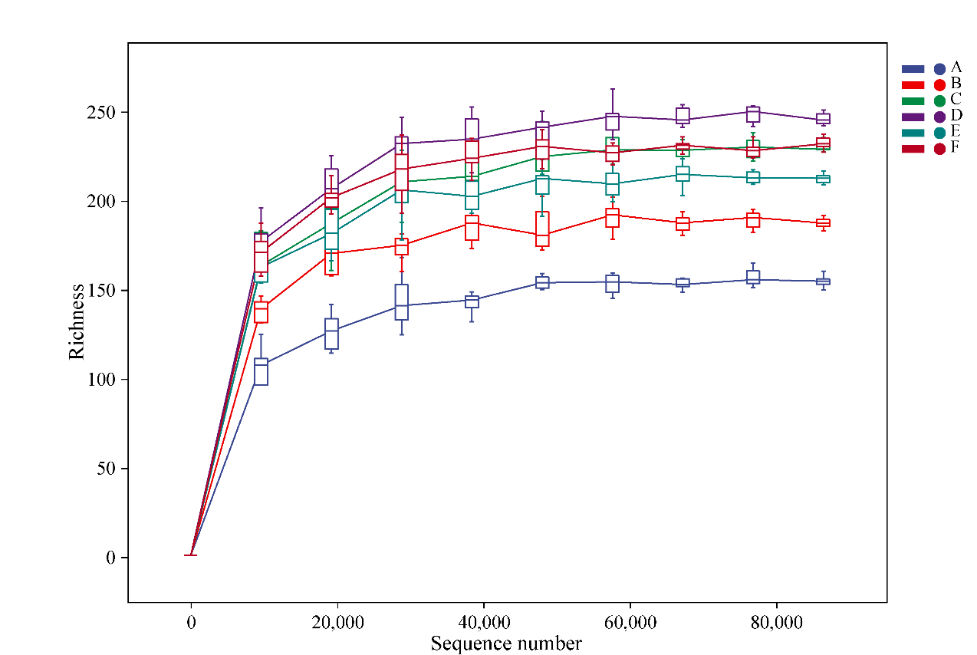


## Figure S2. Assessment of sparse curves for bacterial sequencing saturation.


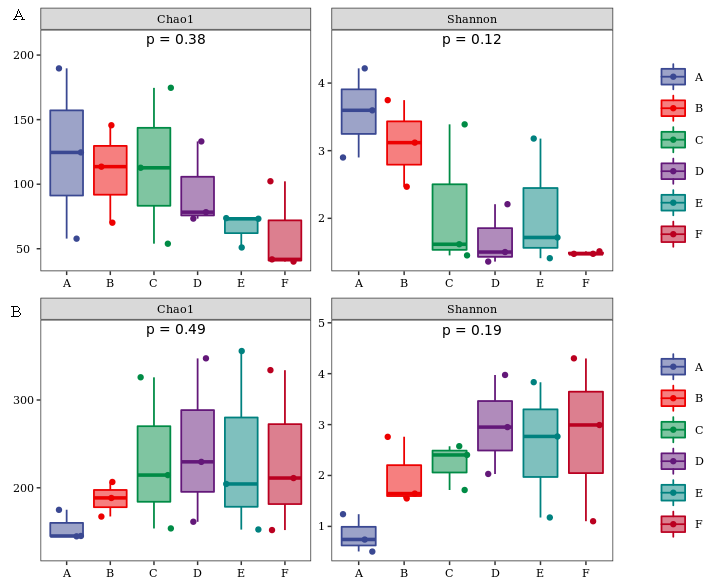


## Figure S3. Alpha diversity indexes (Chao 1 and Shannon) for fungal (A) and bacterial communities (B).


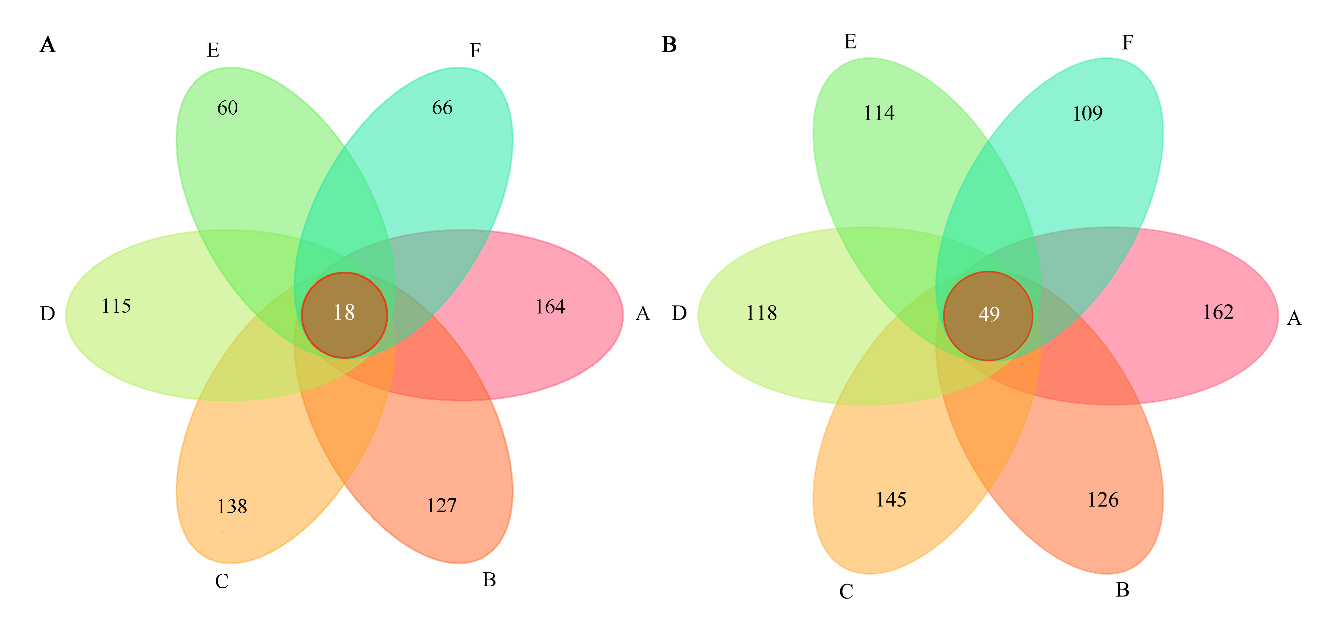


## Figure S4. Venn diagram of fungal (A) and bacterial (B) OTUs among samples.


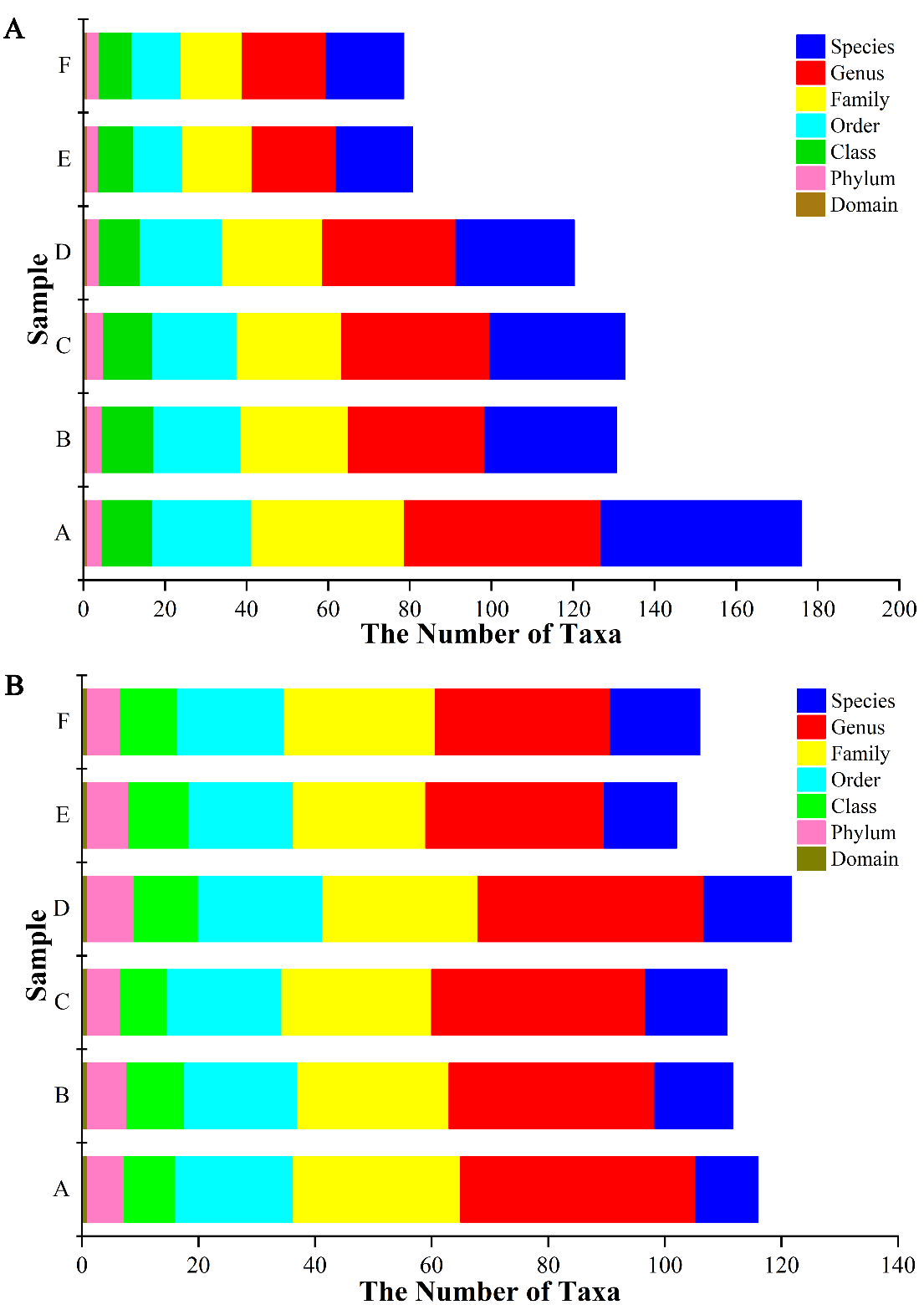


## Figure S5. Statistics of the microbiota at each classification level in fungi (A) and bacteria (B).


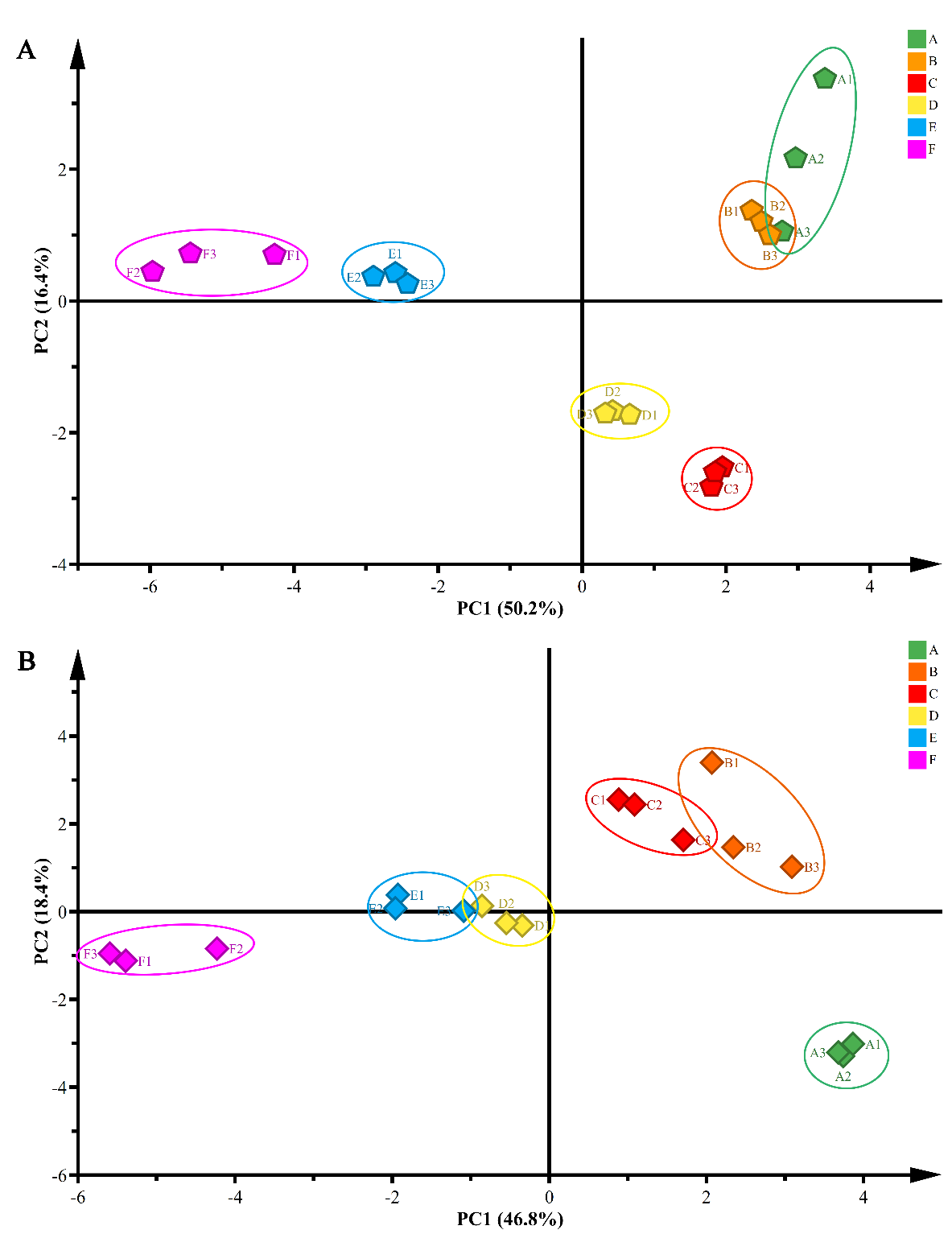


## Figure S6. Principal component analysis (PCA) scatter plot of fungal (A) and bacterial communities (B) in the samples.


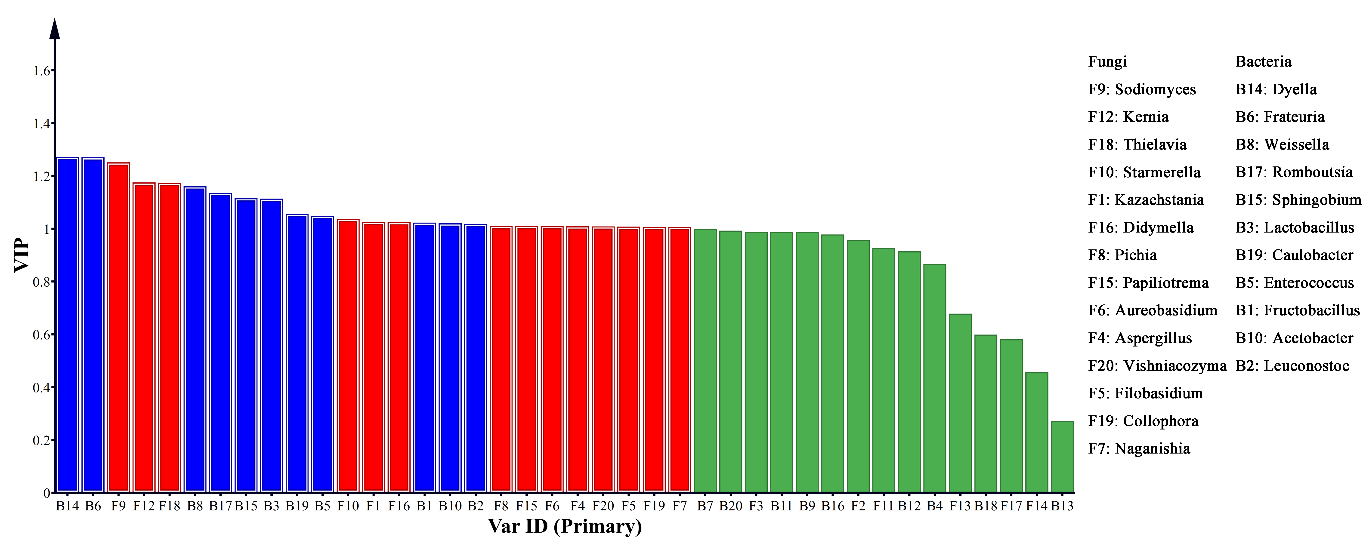


## Figure S7. Variable importance of predictive components (VIP [pred]) plot for the microbiota. The red, blue, and green columns refer to fungi (VIP [pred] > 1), bacteria (VIP [pred] > 1), and microbes (VIP [pred] < 1), respectively.


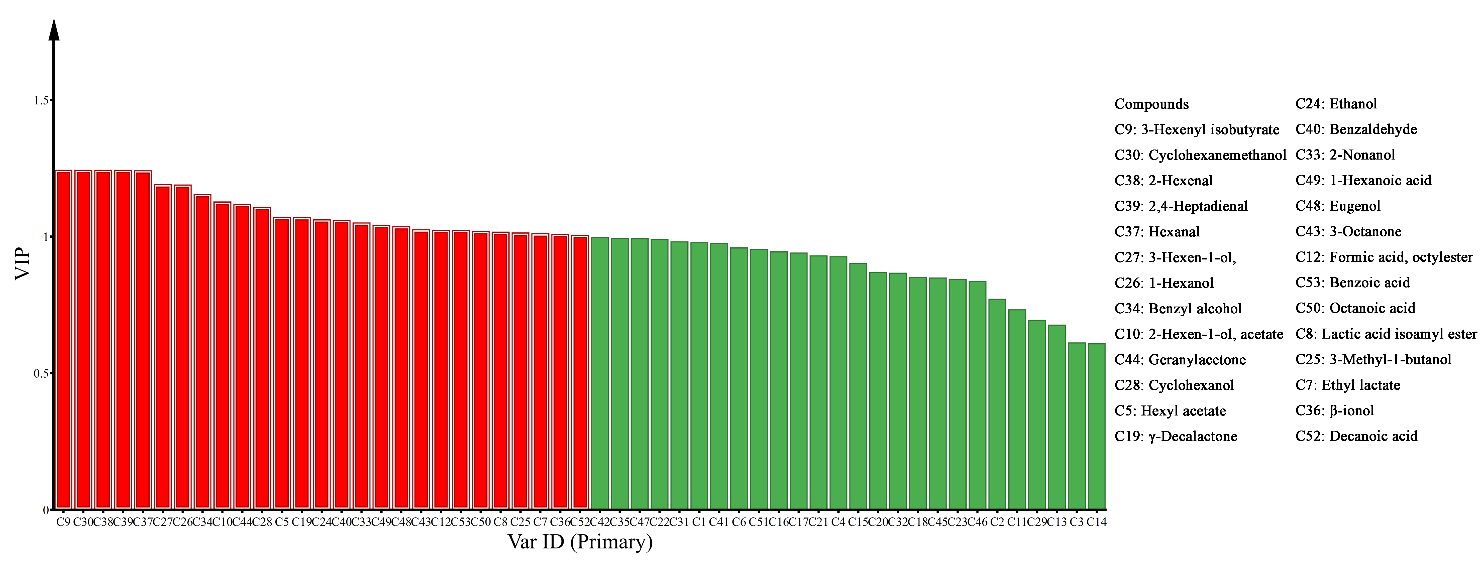


## Figure S8. Variable importance of predictive components (VIP [pred]) plot for volatile compounds.
